# Antibacterial Activity of Copper Nanoparticles against *Xanthomonas campestris* pv. *vesicatoria* in Tomato Plants

**DOI:** 10.3390/ijms23084080

**Published:** 2022-04-07

**Authors:** Adamantia Varympopi, Anastasia Dimopoulou, Dimitris Papafotis, Pavlos Avramidis, Ioannis Sarris, Theodora Karamanidou, Alexandra Kaldeli Kerou, Afroditi Vlachou, Eleftherios Vellis, Andreas Giannopoulos, Kosmas Haralampidis, Ioannis Theologidis, Dimitris G. Hatzinikolaou, Alexander Tsouknidas, Nicholas Skandalis

**Affiliations:** 1 Institute of Molecular Biology and Biotechnology, FORTH, 71110 Heraklion, Greece; avarympopi@biol.uoa.gr (A.V.); anastasia_dimopoulou@imbb.forth.gr (A.D.); 2 Enzyme and Microbial Biotechnology Unit, Faculty of Biology, National and Kapodistrian University of Athens, Zografou, 15784 Athens, Greece; dpapafotis@biol.uoa.gr (D.P.); dhatzini@biol.uoa.gr (D.G.H.); 3 Department of Geology, University of Patras, 26504 Patras, Greece; p.avramidis@upatras.gr (P.A.); insarris@upatras.gr (I.S.); 4 PLiN Nanotechnology S.A., Spectra Business Center 12th km Thessaloniki-Chalkidiki, Thermi, 57001 Thessaloniki, Greece; tk@plin-nanotechnology.com (T.K.); ak@plin-nanotechnology.com (A.K.K.); av@plin-nanotechnology.com (A.V.); at@plin-nanotechnology.com (A.T.); 5 K&N Efthymiadis Single Member S.A., Business Development Division, Industrial Area of Thessaloniki, POB 48, Sindos, 57022 Thessaloniki, Greece; l.vellis@efthymiadis.gr (E.V.); a.giannopoulos@efthymiadis.gr (A.G.); 6 Department of Botany, Faculty of Biology, National and Kapodistrian University of Athens, Zografou, 15784 Athens, Greece; kharalamp@biol.uoa.gr; 7 Laboratory of Pesticides’ Toxicology, Benaki Phytopathological Institute, 14561 Athens, Greece; i.theologidis@bpi.gr; 8 Keck School of Medicine, Health Sciences Campus, University of Southern California, 1441 Eastlake Ave, Los Angeles, CA 90033, USA

**Keywords:** green synthesis, copper bioavailability, *Xanthomonas campestris* pv. *vesicatoria*

## Abstract

Copper-based bactericides have appeared as a new tool in crop protection and offer an effective solution to combat bacterial resistance. In this work, two copper nanoparticle products that were previously synthesized and evaluated against major bacterial and fungal pathogens were tested on their ability to control the bacterial spot disease of tomato. Growth of *Xanthomonas campestris* pv. *vesicatoria*, the causal agent of the disease, was significantly suppressed by both nanoparticles, which had superior function compared to conventional commercial formulations of copper. X-ray fluorescence spectrometry measurements in tomato leaves revealed that bioavailability of copper is superior in the case of nanoparticles compared to conventional formulations and is dependent on synthesis rather than size. This is the first report correlating bioavailability of copper to nanoparticle efficacy.

## 1. Introduction

Copper bactericides and fungicides have been used worldwide in mass volumes in order to protect crop production for over a century [1]. However, due to their poor adhesion to plant tissues, large quantities of this trace element are transferred to water bodies and sediments [2,3]. This is because the existing commercial copper products are based on insoluble forms of copper (II) hydroxide, copper (II) oxide or copper (II) oxychloride in micron-metallic size [4]. 

Nanotechnology is currently focused on the development of innovative antimicrobials, with substantial success and potential application in many fields [5,6,7]. At present, copper-based nanoparticles (CuNPs) have been studied as possible substitutes to commercially available bactericides [8,9]. Several researchers have pointed that the decreased size of metallic particles increases the antibacterial action of the given counterpart [9]. As a result, nanometer-sized compounds are more effective than micron-sized products [10,11]. In fact, their increased activity is based on the small size and high surface to volume ratio, which is reported to contribute to easier membrane penetration and higher metal ion production into solution [9,12]. Several methods have been developed for production of Cu-based NPs [13,14]; however, the small range size and strong stability remains a challenge [15]. For instance, copper hydroxide NPs seem to be metastable, transitioning into copper oxide NPs [16], with pH-dependent stability [17]. In numerous studies, nanoparticles (1–100 nm) have been evaluated as antibacterial agents towards a wide range of bacteria [18,19,20,21], including plant pathogens such as *Ralstonia solanacearum* and *Pseudomonas* spp. [5,22]. Hence, nano-based composites can constitute a new approach in plant protection controlling bacterial diseases. 

Previously published work of our team presented the synthesis of two stable, small-sized products, the first CuNP1 (100% CuO) and the other CuNP2 a mixture of Cu_2_O (64%) and Cu(OH)_2_ (36%), with no phytotoxicity effect [15,23], and their antibacterial activity against seven economically important phytopathogens in vitro [15]. The aim of this study is to explore their potential use *in planta* against *Xanthomonas campestris* pv. *vesicatoria* (*Xcv*), the causative agent of the bacterial leaf spot disease of tomato. *Xcv* was chosen as a representative species with high occurrence of antibiotic- and copper-resistant isolates [24,25,26]. Recently, independent studies of Cu-based NPs of different sizes have shown significant control of the bacterial leaf spot compared to conventional bactericides in the field [8,9,27]. However, nanoparticle-type-dependent bioavailability and mode of action, which largely determine *in planta* efficacy, remain understudied. In this work, we displayed (i) an in vitro and *in planta* evaluation of CuNPs against *Xanthomonas campestris* pv. *vesicatoria*, (ii) a comparison of effectiveness between CuNPs and conventional copper oxide/hydroxide products and (iii) a correlation of bioavailability of copper with its antibacterial efficacy. Additionally, for the first time X-ray fluorescence spectrometry (XRF) was used to measure the adhesion of copper elements of CuNP products. 

## 2. Results

### 2.1. Dose–Response Effect of Copper Nanoparticles against Xanthomonas campestris pv. vesicatoria

**Figure 1:**
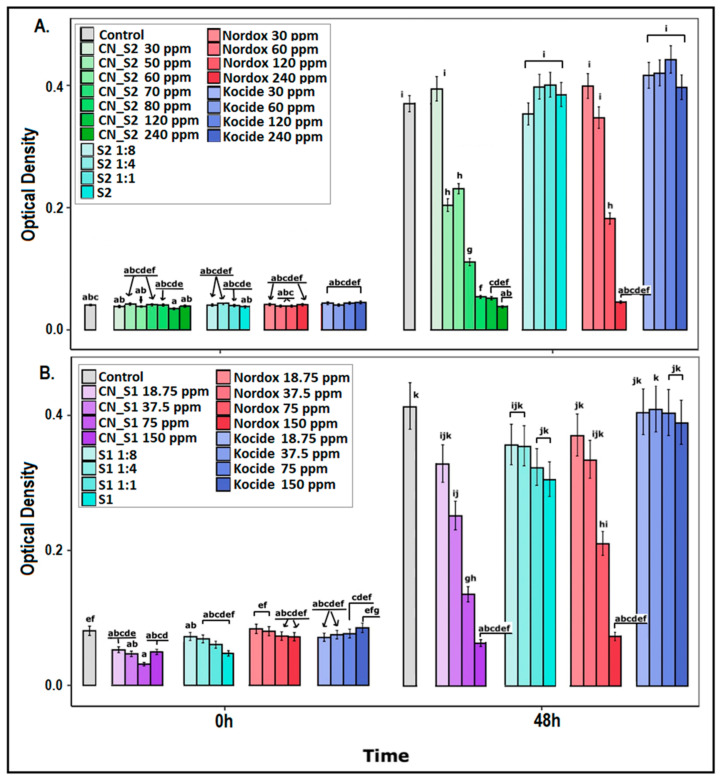
Dose–response effect of two copper nanoparticles at increasing concentrations against the bacterial pathogen *X. c.* pv. *Vesicatoria*: (**A**) CN_S2, CN_S2_X1 and (**B**) CN_S1, CN_S1_X1. Respective stabilizers S1 and S2 and water treatment were used as controls, while Nordox and Kocide were used as reference compounds. Effect was evaluated in OD 600 at 0, 24 and 48 h post inoculation (hpi) using a multi-detection microplate reader. Estimated marginal means and their standard errors for three independent experiments of triplicate data sets are plotted here. Different letters (a–k) represent statistically different data points at *p* ≤ 0.05 according to Tukey post hoc comparisons. Comparisons are between all treatments and time points. Brackets and arrows indicate bars in the same statistical group.

*Xanthomonas campestris* pv. *vesicatoria* susceptibility was assessed against increasing concentrations of CN_S1 (CuNP1) and CN_S2 (CuNP2), for 48 h. The full results are depicted in Supplementary Figure S1, while the results of the 48 h are depicted here (Figure 1A,B) for simplicity and to avoid confusion. MIC calculations at 48 hpi are shown in Table 1. Active ingredient stabilizers, nominated as S1 and S2, respectively, and water were used as controls. Nordox and Kocide were used as reference copper compounds at equal increasing concentrations. Both copper nanoparticles were found to be effective against *X. c.* pv. *vesicatoria* and statistical comparisons to controls were significant (Figure 1A,B). Most importantly, CuNPs were more effective compared to the reference treatments.

In particular, *X. c.* pv. *vesicatoria* was found to be equally as susceptible to CN_S2 as and Nordox; MIC concentration at 48 h post inoculation (hpi) was calculated to be >120 ppm in both cases (Figure 1A, Table 1). As the concentration decreased, a significant difference between the two was observed, with CN_S2 displaying higher efficiency; its effective range spanned as low as 50 ppm compared to 120 ppm for Nordox. This delay allowed for MIC50 and MIC90 to be calculated at 64 and 240 ppm, respectively (Figure S1). Respective calculations were 10 and 180 ppm in the case of Nordox. 

In the case of CN_S1, increased susceptibility of *X. c.* pv. *vesicatoria* was confirmed in dose–response experiments compared to Nordox but also CN_S2 (Figure 1B, Table 1). MIC, MIC50 and MIC90 calculations >75, =90 and =130 ppm, respectively, were significantly lower compared to CN_S2. Similar effects were observed for Nordox (>120 ppm, =100 ppm, =140 ppm) and CN_S2 (as mentioned above). The range concentration (concentration in which a significant effect occurs compared to control) was observed at 37.5 ppm for CN_S1 but at 75 ppm for Nordox. 

In both CuNPs (CN_S2 and CN_S1) and Nordox, the Minimum Bactericidal Concentration (MBC) experiments at MIC concentrations suggested a bacteriostatic effect instead of bactericidal. Surprisingly, Kocide had no effect in all tested concentrations. Stabilizers S1 and S2 had no effect, as expected.

### 2.2. Efficacy of CN_S2 and CN_S1 against the Bacterial Spot of Tomato

**Figure 2:**
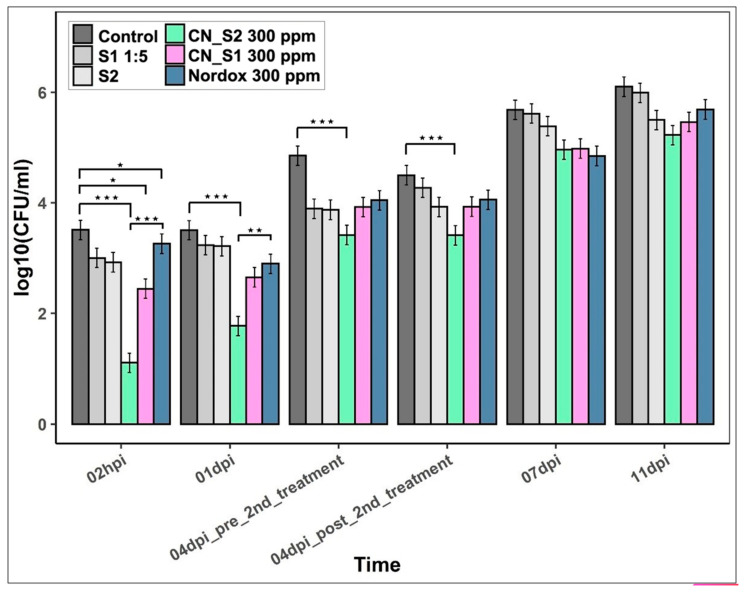
*X. campestris* pv. *vesicatoria* population growth in tomato leaves sprayed with the two copper nanoparticles (CN_S2 and CN_S1), their stabilizers S2 and S1 and Nordox at concentration of 300 ppm. Estimated means and corresponding standard errors of the logarithm of the bacterial population from 4 samples are shown. *: *p* < 0.05; **: *p* < 0.01; ***: *p* < 0.001 according to Tukey post hoc comparisons.

The *in planta* efficacies of CN_S2 and CN_S1 were based on the *X. c*. pv. *vesicatoria* total population in leaves of tomato plants sprayed with each CuNP over a period of 11 days post inoculation (dpi) (Figure 2). Inoculation was applied by spraying to develop infection (Supplementary Figure S2). Nordox was used as the reference standard, while stabilizers (S2 and S1) and water were used as controls. Both CN_S2 and CN_S1 were found to significantly reduce the total population of *X. c.* pv. *vesicatoria* until 4 dpi compared to control treatment, while at 7 and 11 dpi the respective drop in internal population density was not statistically significant. The highest difference between CN_S2 and the control treatments was observed at 2 hpi, suggesting acute toxicity of CN_S2 against the pathogen and its establishment on the host. Similarly, CN_S2 showed a significant difference from Nordox which persisted until 1 dpi, suggesting a clear advantage during the primary infection. CN_S1 was found to be less effective compared to CN_S2 and Nordox at 2 hpi and 1 dpi but still effective compared to controls. At 4 dpi, a second spraying occurred, which according to pre and post time points had no effect on the population of *X. c.* pv. *vesicatoria* at all treatments. Finally, stabilizers S1 and S2 had no effect on pathogen infection compared to water treatment at all time points.

### 2.3. Total Copper Concentration on Tomato Leaves

**Figure 3:**
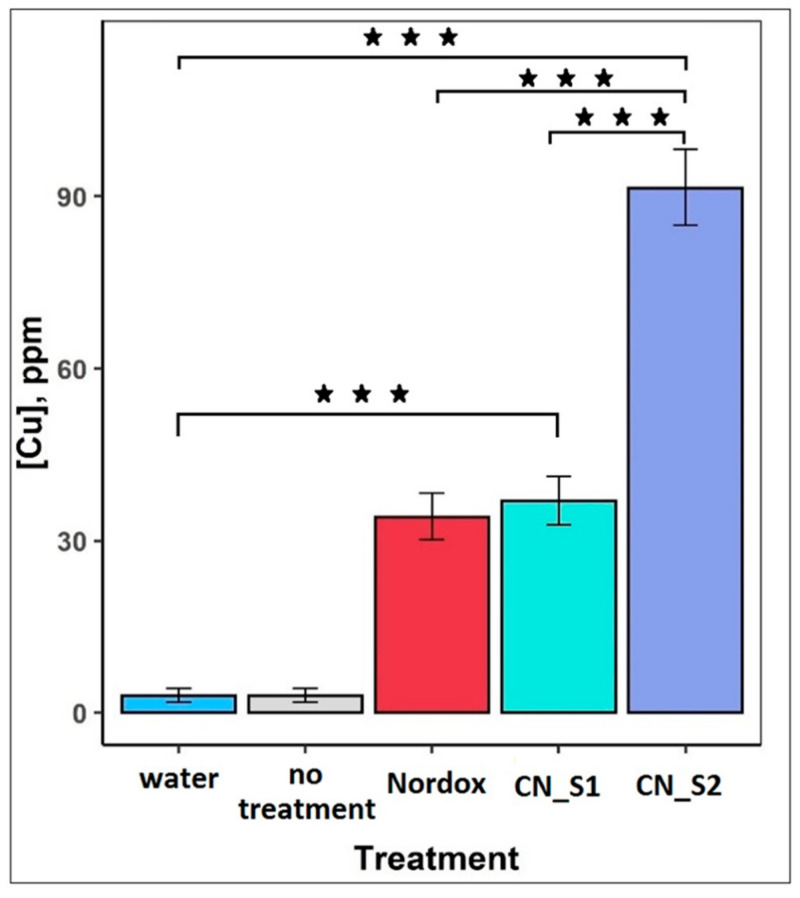
X-ray fluorescence spectrometry of copper concentration on tomato leaves after sprayed with the two copper nanoparticles (CN_S1 and CN_S2) at a concentration of 300 ppm. Water and Nordox (300 ppm) were used as control and standard reference, respectively. Estimated marginal means and standard errors of tomato leaves copper concentration from 15 plants per treatment are plotted. ***: *p* < 0.001 according to Tukey post hoc comparisons.

To measure the available total copper concentration on the surface of tomato leaves an X-ray fluorescence spectrometry method (XRF) was used. Results are depicted in Figure 3. Water and no treatment led to a barely detectable (4.25 ppm) copper concentration, which defined the natural occurrence of copper in plant tissue. The highest copper concentration was observed in the CN_S2 treatment and measured at 90 ppm. CN_S1 and Nordox showed the same levels of copper concentration at 40.8 and 35.35 ppm, respectively. CN_S2 bioavailability on the tomato leaves was significantly higher compared to that of Nordox and CN_S1.

## 3. Discussion

Bacterial plant diseases, less multitudinous than fungal diseases, are considered challenging to control, due to their frequent polycyclic nature and the limitations of systemic and environmentally friendly antibacterial substances [28,29,30]. The existing list of conventional products containing copper needs to be diminished because of major concerns of residues in food and water and its negative environmental impact [31,32]. Furthermore, *Xanthomonas* species are remarkably successful in developing resistant populations due to excessive use of copper compounds and antibiotics [33,34,35]. Alternative compounds have shown to be promising but are usually used synergistically with conventional copper [36]. Therefore, a more sustainable and effective approach is needed to tackle devastating pathogens such as *X. campestris* pv. *vesicatoria*. 

In the present study, our in vitro results demonstrated that CuNPs had an effective antimicrobial action against *X. campestris* pv. *vesicatoria*. CN_S1 showed superior performance compared to CN_S2 but also conventional copper products Nordox and Kocide. This confirms previous findings: CN_S1, a Cu_2_O-based product, was found to be overall more effective compared to CN_S2 and conventional copper products in restraining growth of several tested plant pathogenic bacteria and fungi [15,23]. 

The results of the *in planta* trials strongly suggest that CN_S2 exhibited a superior performance compared with Nordox, a successful commercial product, in respective concentrations. To the best of our knowledge, there is a scarcity of studies with evaluation of copper nanoparticles *in planta* because of their high phytotoxicity [8,37]. As we have mentioned in previous work, CuNPs had no toxic effect in tomato leaves sprayed with concentrations equal or higher to MIC [15]. The lower bacterial population was achieved with applications at a rate of 300 µg/mL, which was significantly lower than the commercially recommended rates of 1120 µg/mL for the copper oxide product. This evidence shows that CuNPs with lower concentrations of Cu are efficient to control bacterial leaf spot, in significantly lower concentrations than those used in practice in the case of conventional compounds. This suggests that use of CuNPs can diminish the adverse effects of copper on the environment and soil biota [38,39]. Antimicrobial activity of copper nanoparticles, which has been observed from other studies, are in accordance with our results. In particular, concentrations of 100–250 ppm have been shown effective against several bacterial species such as *Ralstonia*, *Pseudomonas* and *Xanthomonas,* in vitro and *in planta* [5,9,22,40,41], while similarly, silver nanoparticles have also exhibited strong antibacterial action against *Xanthomonas* spp. [42,43,44]. CN_S1 and CN_S2 have exhibited a size of 5.23 ± 0.8 and 10.41 ± 1.2, respectively [14], significantly smaller than others that have been previously mentioned. Antibacterial activity of copper nanoparticles is expected to be superior in the case of small size and high surface–volume ratio [12,45].

The two-day pre-inoculation application of CN_S2 statistically differentiates the pathogen’s establishment from Nordox and water treatment (Figure 2). The second application six days post inoculation did not reduce the bacterial population, suggesting that CN_S2 action is protective but not therapeutic. This has also been suggested in a previous study, where the protective activity of the two CuNPs was evaluated against *Fusicladium oleagineum* and *Colletotrichum* spp. in olive trees [23].

CN _S2 MIC was calculated higher than CN_S1 (Table 1); however, *in planta* study, CN_S2 displayed superior efficacy (Figure 2). The X-ray fluorescence spectrometry measurements, which revealed higher available copper concentration in the case of CN_S2 application, and consequently a higher bioavailability, suggest that CuNPs’ mode of synthesis and formulation may play an important role not only for antimicrobial susceptibility and phytotoxicity, but also for attachment to plant surfaces. Attachment and bioavailability are considered as major determinants of nanoparticle efficacy [46]. CuNPs affect the surface charge and the hydrophobicity of activated sludge, which plays an important role in the attachment of bacteria to the surface [47]. Further to accumulation, bioavailability is also affected by water aggregation in spraying solutions due to the hydrophobic nature of copper hydroxide and copper oxide particles. Reduced bioavailability results in lower control efficacy [45]. The two different types of CuNPs were designed to minimize aggravation, with the lowest possible size and higher negative charge to augment their activity. Our field experience suggests that in field studies efficacy is based on symptom development. All commercial formulations practically fail to eradicate pathogens, and there are still pathogen counts. However, the reduction of primary inoculations due to a reduction of initial populations can reduce following outbreaks. Therefore, we believe that our results are relevant to real-life conditions, where protective applications occur weakly.

Despite the fact that several modes of action have been suggested for metallic nanoparticle-based bactericides, there is a consensus in the literature that most of them relate to complex-forming reactions of their ions [46]. Taken together with the composition of both CN_S1 and CN_S2 used in this study, featuring 100% NPs and no ions, it stands to reason that the CuNPs function as an ion reservoir, gradually releasing ions that permeate the cellular membrane, inhibiting their replication and/or disrupting other cellular functions through the generation of reactive oxygen species.

As a result of this, NP-based formulations have a much more controlled ion release than other copper-based bactericides that instantly dissolve into ions. This could explain the superior antibacterial performance of CuNPs over prolonged periods, whereas their immediate efficacy is likely attributed to their small size, allowing for a favorable dispersion/coverage.

## 4. Materials and Methods

### 4.1. CuNP Characteristics

As was previously presented and confirmed by TEM imaging, the two types of CuNPs exhibited a population of monodispersed NPs, with average diameter between 5.10 and 10.41 nm [23]. TEM images of the CuNPs also confirmed that both types have spherical morphology, with an average size between 5 and 10 nm [23]. Lastly, XPS data previously published by our group indicate that the CN_S1 is 100% cupric oxide (CuO), whereas the CN_S2 is a mixture of cuprous oxide (Cu_2_O) and copper hydroxide (Cu(OH)_2_) in a ratio of 64% and 36%, respectively [15]. 

### 4.2. Synthesis and Characterization of CuNPs

Nanoparticles were synthesized based on a wet chemistry approach, as described in Varympopi et al. [15]. In brief, the precursor salt for both CN_S1 and CN_S2 (copper (II) nitrate purchased from Alfa Aesar) was magnetically stirred for 15 min in deionized water, while maintaining a pH of 10–11, using 0.5 M of sodium hydroxide (purchased from CHEM-LAB). CN_S1 was produced through the gradual addition of the copper salt to stabilizer S1 (an animal protein purchased from Sigma Aldrich, St. Louis, Missouri), while CN_S2 was formed through the rapid addition to stabilizer S2 (a non-ionic polymer purchased from Alfa Aesar). The process was considered as complete once the color of the solution changed to purple and bright green for CN_S1 and CN_S2, respectively.

A Tangential Flow Filtration (TFF) method was employed in order to evaluate the yield of the reduction process, as has been previously used by Platania et al. [43]. During this process, the colloidal dispersion of NPs flows tubularly through a membrane. Anything smaller in size than the membrane’s pores can permeate (i.e., organic constituents or Cu ions). Following this, the copper content of the supernatant solution (i.e., high in copper ion content) and the content of the retentate solution containing the CuNPs, was evaluated through Inductively Coupled Plasma-Optical Emission Spectrometry (ICP-OES). 

### 4.3. Bacterial Strain and Growth Conditions

Rifampicin-resistant *Xanthomonas campestris* pv. *vesicatoria* (*Xcv*) strain 85-10 (isolated from pepper, GenBank accession number AM039952 [44]) was used throughout the experimental procedure, routinely grown at 28 °C in Luria–Bertani (LB) broth or on LB agar medium with 100 μg/mL rifampicin added. 

### 4.4. Broth Microdilution Method

*X. campestris* pv. *vesicatoria* strain 85-10 was grown exponentially in overnight cultures and was checked for culture purity by streaking on selective growth medium. The final concentration of culture aliquots was adjusted to 5 × 10^6^ cfu/mL. The implemented method that was followed was described by Varympopi et al. [15]. In brief, each microtiter plate containing (Greiner CELLSTAR 96-well microplates F-bottom) a single CuNP (CN_S1_X1 and CN_S2_X1), its stabilizer (S1 and S2) and the two reference copper-based commercial products Kocide OPTI 30 WG (Kocide LLC,12701 Almeda Road, Houston TX 77045 USA) and Nordox 75 WP (NORDOX Industrier As, Ostensjoveien 13, OSLO 6, Norway) in the same concentrations and control was incubated at 28 °C and 200 rpm for 48 h. Three independent biological experiments were performed for each CuNP. Optical Density (OD) absorbance (600 nm) measurements were held at 0, 24 and 48 h. The effect of CuNPs on bacterial growth was assessed by determining Minimum Inhibitory Concentration (MIC) MIC90 and MIC50 and Minimum Bactericidal Concentration (MBC) [25]. 

### 4.5. Plant Growth Conditions

Tomato (*Solanum lycopersicum* cultivar “Moneymaker”) seeds were pre-germinated on moist potting soil under controlled conditions (25 °C and 16 h photoperiod). After 10 days, plantlets were transplanted into 9 cm square pots filled with a three-element (N-K-P) fertilizer compost and were left to grow until the developmental stage of four true leaves before starting with experiments. 

### 4.6. Pathogen Inoculum Preparation

To prepare the bacterial inoculum, a pre-culture of *Xcv* was grown o/n in 5 mL of liquid LB medium at 30 °C and 200 rpm. LB medium (200 mL) was inoculated with 0.16 mL of the *X. campestris* pv. *vesicatoria* pre-culture and incubated at the same conditions until an OD600 of 0.7; bacterial cells were then harvested by centrifugation at 2.800 rpm for 10 min and re-suspended at an OD600 of 0.12 (10^8^ colony forming units (cfu) mL^−1^) in sterile water. Spray inoculation was employed to simulate natural infection of tomato plants [48]. Leaves were gently wounded before sprayed until run-off, using a hand-held pneumatic sprayer. Plants were then allowed to dry until no droplet was visible and returned to the incubation area.

### 4.7. CuNP Application, Design and Layout of Bacterial Infection Experiments

CN_S1 and CN_S2 were synthesized by PLiN Nanotechnology and used in a concentration of 300 ppm which according to in vitro experiments is higher than MICs and is the maximum possible concentration of the composition of the CN_S2. Stabilizers, lacking the active nanoparticles and Nordox, were used in the same concentration as blank formulation and reference standard for bacterial disease assays, respectively. Water was used as control. Nordox was selected in favor of Kocide due to its high toxicity according to in vitro experiments. Plants were sprayed until run-off using a 5L calibrated commercial sprayer in a 7-day interval with the first application to be performed 2 days before inoculation. Plants were then allowed to dry until no droplet was visible and returned to the incubation area. Each treatment plot included 9 plants and experiments were repeated three times. Sampling was performed at seven time points post inoculation (dpi) (2 hpi, 1 dpi, 4 dpi-pre 2nd application, 4 dpi-post 2nd application, 7 dpi and 11 dpi) and every sample was a pool of 3 leaf discs of 3 different plants (3 samples per treatment). Samples were homogenized with tissue homogenizer pestle in 1.5 mL Eppendorf tubes containing 1 mL of sterile distilled water and 30% glycerol. Aliquots of 0.1 mL from a 10-fold dilution series were plated on nutrient agar (NA) medium supplemented with 5 μg/mL rifampicin, incubated for 48 h at 28 °C and pathogen population was estimated as previously described [49] by cell counts. Infection and bacterial spot disease development was based on population growth to avoid bias and misinterpretation of symptoms due to wounding of leaves.

### 4.8. X-ray Fluorescence Spectrometry (XRF) for Copper Measurement of CuNP Products in Tomato Leaf Tissue

Tomato plants of four true leaves in the developmental stage were sprayed as described above with the two copper nanoparticles (CN_S1 and CN_S2) and Nordox in a concentration of 300 ppm. Water was used as control while a non-treated plot was added as control for the copper traces of water treatment. Plants were then allowed to dry until no droplet was visible and transferred at 60 °C for 24 h. 

A non-destructive method, X-ray fluorescence (XRF) spectrometry, was used to determine the overall elemental analysis of the leaf samples and for the semi-quantitative measurement of copper on their surface, using portable energy-dispersed X-ray fluorescence (ED-XRF) BRUKER S1 Titan 800 equipment. During the measurement process, the procedures described in the International Standard ISO 13196: 2013 were followed. The analyses took place in the Laboratory of Sedimentology, Department of Geology, University of Patras. which is accredited according to ISO 17025/17 for this type of analysis. Prior to analysis, the instrument manufacturer’s instructions for adjusting, cleaning and preparing were followed. In addition, performance and stability of the instrument were tested using multi-component reference samples.

In each leaf sample, multiple measurements were taken (10 measurements at different points of its upper and lower surface), with a disk control surface of 5 mm diameter per position and operating conditions of the X-ray generator 15 kV @ 12 uA Rh anode without filters, and Detector Type Fast SDD with resolution <145 eV. 

### 4.9. Data Analysis

#### 4.9.1. Broth Microdilution Method 

Measurements for each species were collected in triplicated 96-well plates, which were considered as different experimental blocks. Log10 of OD measurements was modeled using Linear Mixed Effects Models (LMMs). Time, treatment (concentration of substance used) and their interaction were the fixed factors, while sample by experiment comprised the random part of the model. Blanks were modeled beforehand under the same strategy and the estimates of each treatment combination were subtracted from the corresponding experimental ODs, a fact that reduced initial values towards zero. Estimated marginal means were obtained by the emmeans function of the emmeans package in R (Russell V. Lenth (2021). Emmeans: Estimated Marginal Means, also known as Least-Squares Means. R package version 1.7.0. https://CRAN.R-project.org/package=emmeans, accessed on 10 July 2021). Tukey tests were applied for post hoc comparisons of Estimated Marginal Means and back-transformed values were plotted. Minimal Inhibitory Concentrations (MICs) (MIC90, MIC50) were measured in statistical language R as described in Skandalis et al. [49]. 

#### 4.9.2. Efficacy Evaluation Experiments

Pathogen growth in planta was assessed as previously described [18,36,48]. Cfu counts were modeled using LMMs to log10 transformed data (number of bacterial cfu) for each time point. Time after inoculation and treatment, as well as their interaction, were modeled as fixed factors, while plant individuals nested within the block comprised the random factor. Tukey tests were applied for post hoc comparisons of estimated marginal means.

#### 4.9.3. X-ray Spectrometry Experiments

X-ray fluorescence spectrometry of copper concentration was modeled using a Linear Model approach. Square rooted values of [Cu] were the dependent variables, while treatment was the independent variable of the model. Tukey tests were applied for comparisons and back-transformed values were plotted.

## 5. Conclusions

In this work, the two developed CuNPs were evaluated against *X. campestris* pv. *vesicatoria*, the causative agent of bacterial leaf spot, in vitro and *in planta*. Both showed higher bactericidal activity compared to their respective commercial copper products. CN_S2 demonstrated as effective to prevent the establishment of *Xcv* in low concentrations. XRF measurements indicate that nanotechnology increases the bioavailability of copper in plant tissue compared to conventional copper bactericides, and allows production of effective, stable and environmentally friendly compounds for crop protection. 

## Figures and Tables

**Table 1:** Calculated MICs and MBCs of broth efficacy tests of CuNPs for *X. c.* pv. *vesicatoria*.

	CN_S1 (ppm)	CN_S2 (ppm)	Nordox (ppm)	Kocide (ppm)	S1	S2
**MIC**	>75	>120	>120	>240	-	-
**MIC50**	90	64	100 ^a^/110 ^b^	NA	-	-
**MIC90**	130	240	140 ^a^/180 ^b^	NA	-	-
**MBC**	>150	>240	>240	>240	-	-

^a^ CN_S2, ^b^ CN_S1.

## Supplementary Materials

The following supporting information can be downloaded at: https://www.mdpi.com/article/10.3390/ijms23084080/s1.
