# Supplementary material for: Antibacterial Activity of Copper Nanoparticles against Xanthomonas campestris pv. vesicatoria in Tomato Plants
Source: Int J Mol Sci. 2022 Apr 7;23(8):4080. doi: 10.3390/ijms23084080 (PMC9032352; doi:10.3390/ijms23084080)
Supplement: Supplementary file 1 [file ijms-23-04080-s001.zip › ijms-1663346-supplementary.pdf]

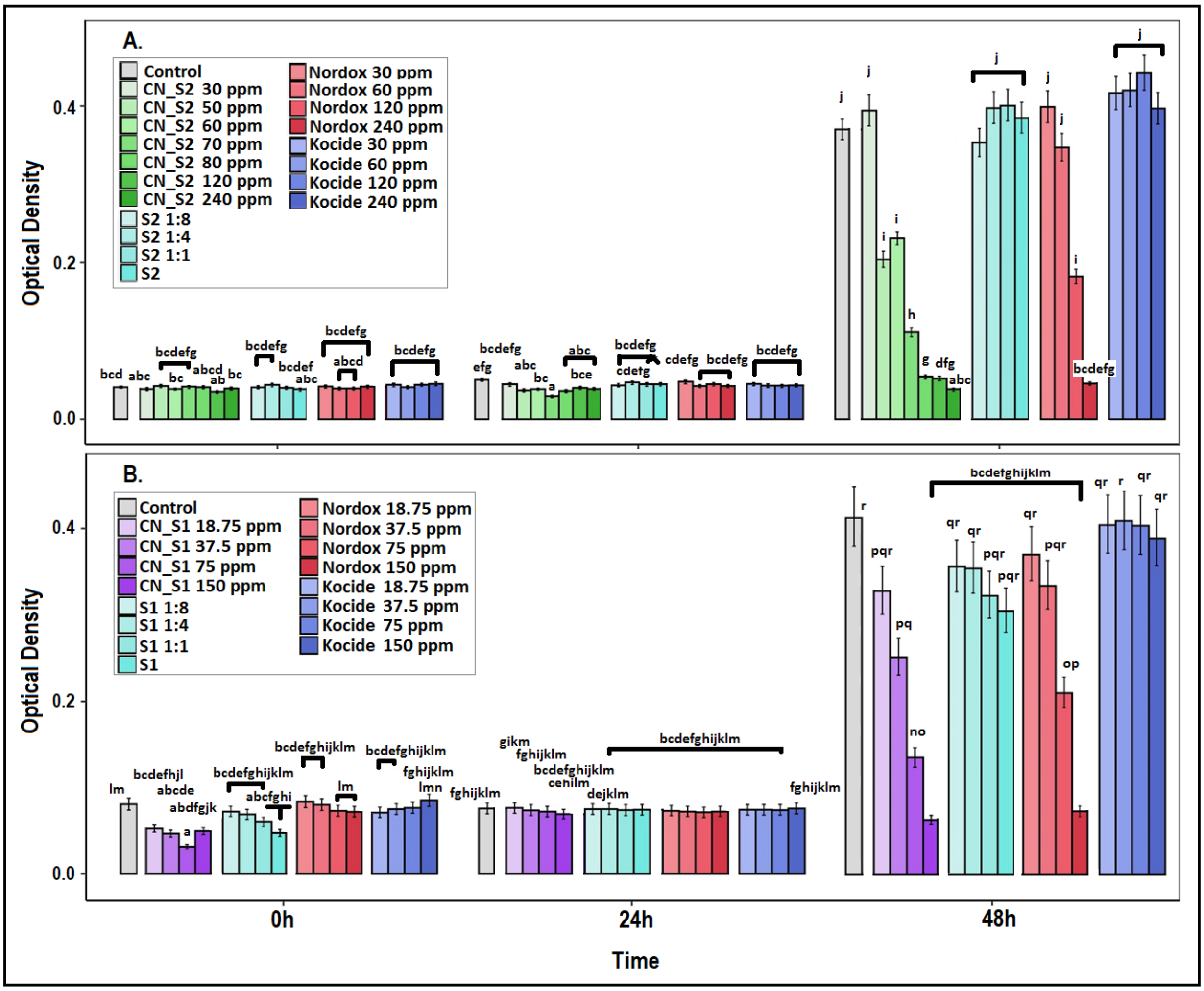

Figure S1. Dose–response effect of two copper nanoparticles at increasing concentrations against the bacterial pathogen *X. c.* pv. *vesicatoria*; A) CN_S2, CN_S2_X1 and B) CN_S1, CN_S1_X1. Respective stabilizers S1 and S2 and water treatment were used as controls, while Nordox and Kocide were used as reference compounds. Effect is evaluated in OD 600 at 0, 24 and 48 h post inoculation (hpi) using a multi-detection microplate reader. Estimated marginal means and their standard errors for three independent experiments of triplicate data sets are plotted here. Different letters (a-r) represent statistically different data points at P ≤ 0.05 according to Tukey post hoc comparisons.

FigureS1_A. Comparisons between all treatments through time (CN_S2_X1)							
times	treatment	emmean[1]	SE[2]	df[3]	lower.CL[4]	upper.CL[5]	group[6]
24h	Green_70	-1,536	0,022	384	-1,61	-1,462	a
0h	Green_120	-1,461	0,022	384	-1,535	-1,387	ab
24h	Green_80	-1,445	0,022	384	-1,519	-1,371	abc
24h	Green_50	-1,437	0,022	384	-1,511	-1,363	abc
48h	Green_240	-1,422	0,0213	493	-1,494	-1,351	abc
0h	S2	-1,422	0,022	384	-1,496	-1,348	abc
24h	Green_60	-1,419	0,0156	384	-1,472	-1,367	bc
0h	Green_30	-1,417	0,022	384	-1,491	-1,343	abc
0h	Green_60	-1,415	0,0156	384	-1,468	-1,363	bc
24h	Green_240	-1,413	0,0208	502	-1,483	-1,343	abc
0h	Green_240	-1,411	0,0209	504	-1,481	-1,34	bc
0h	N_60	-1,41	0,022	384	-1,484	-1,336	abcd
0h	N_120	-1,41	0,022	384	-1,484	-1,336	abcd
24h	Green_120	-1,403	0,022	384	-1,477	-1,329	bce
0h	S2_1:1	-1,402	0,022	384	-1,476	-1,328	bcdef
0h	S2_1:8	-1,393	0,022	384	-1,467	-1,319	bcdefg
0h	Green_80	-1,391	0,022	384	-1,465	-1,317	bcdef
0h	K_60	-1,39	0,022	384	-1,464	-1,316	bcdefg
0h	Mock	-1,389	0,0156	384	-1,442	-1,337	bcd
0h	Green_70	-1,386	0,022	384	-1,46	-1,312	bcdefg
0h	N_240	-1,386	0,022	384	-1,46	-1,312	bcdefg
0h	N_30	-1,38	0,022	384	-1,454	-1,306	bcdefg
24h	N_60	-1,377	0,022	384	-1,451	-1,303	bcdefg
24h	N_240	-1,377	0,022	384	-1,451	-1,303	bcdefg
0h	Green_50	-1,376	0,022	384	-1,45	-1,302	bcdefg
24h	K_120	-1,371	0,022	384	-1,445	-1,297	bcdefg
24h	K_60	-1,369	0,022	384	-1,443	-1,295	bcdefg
24h	K_240	-1,365	0,022	384	-1,439	-1,291	bcdefg
24h	S2_1:8	-1,363	0,022	384	-1,437	-1,289	bcdefg
0h	S2_1:4	-1,361	0,022	384	-1,435	-1,287	bcdefg
0h	K_30	-1,359	0,022	384	-1,433	-1,285	bcdefg
0h	K_120	-1,358	0,022	384	-1,432	-1,284	bcdefg
24h	Green_30	-1,354	0,022	384	-1,428	-1,28	bcdefg
24h	S2_1:1	-1,353	0,022	384	-1,427	-1,279	bcdefg
24h	S2	-1,352	0,022	384	-1,426	-1,278	bcdefg
24h	N_120	-1,35	0,022	384	-1,424	-1,276	bcdefg
24h	K_30	-1,348	0,022	384	-1,422	-1,274	bcdefg
0h	K_240	-1,347	0,022	384	-1,421	-1,273	bcdefg
48h	N_240	-1,341	0,022	384	-1,415	-1,267	bcdefg
24h	S2_1:4	-1,33	0,022	384	-1,404	-1,256	cdefg

24h	N_30	-1,323	0,022	384	-1,397	-1,249	cdefg
24h	Mock	-1,298	0,0156	384	-1,35	-1,245	efg
48h	Green_120	-1,284	0,022	384	-1,358	-1,21	dfg
48h	Green_80	-1,268	0,022	384	-1,342	-1,193	g
48h	Green_70	-0,955	0,022	384	-1,029	-0,881	h
48h	N_120	-0,74	0,022	384	-0,814	-0,666	i
48h	Green_50	-0,69	0,022	384	-0,764	-0,616	i
48h	Green_60	-0,636	0,0156	384	-0,688	-0,584	i
48h	N_60	-0,46	0,022	384	-0,534	-0,386	j
48h	S2_1:8	-0,452	0,022	384	-0,526	-0,378	j
48h	Mock	-0,432	0,0156	384	-0,484	-0,379	j
48h	S2	-0,415	0,022	384	-0,489	-0,341	j
48h	Green_30	-0,404	0,022	384	-0,478	-0,33	j
48h	K_240	-0,401	0,022	384	-0,475	-0,327	j
48h	S2_1:4	-0,401	0,022	384	-0,475	-0,327	j
48h	N_30	-0,399	0,022	384	-0,473	-0,325	j
48h	S2_1:1	-0,397	0,022	384	-0,471	-0,323	j
48h	K_30	-0,381	0,022	384	-0,455	-0,307	j
48h	K_60	-0,377	0,022	384	-0,451	-0,303	j
48h	K_120	-0,355	0,022	384	-0,429	-0,28	j

FigureS1_B. Comparisons between all treatments through time (CN_S1_X1)

times	treatment	emmean	SE	df	lower.CL	upper.CL	.group
0h	Purple_75	-1,499	0,0365	248	-1,62	-1,378	a
0h	Purple_37,5	-1,331	0,0365	248	-1,452	-1,21	abcde
0h	S1	-1,324	0,0365	248	-1,446	-1,203	abcfghi
0h	Purple_150	-1,306	0,0342	319	-1,419	-1,193	abdfgjk
0h	Purple_18,75	-1,279	0,0365	248	-1,4	-1,157	bcdefhjl
0h	S1_1:1	-1,219	0,0365	248	-1,341	-1,098	bcdefghijklm
48h	Purple_150	-1,196	0,0348	320	-1,312	-1,081	bcdefghijklm
0h	S1_1:4	-1,162	0,0365	248	-1,283	-1,04	bcdefghijklm
24h	Purple_150	-1,159	0,034	316	-1,272	-1,046	cehilm
0h	K_18,75	-1,148	0,0365	248	-1,269	-1,026	bcdefghijklm
24h	N_75	-1,147	0,0365	248	-1,268	-1,026	bcdefghijklm
0h	N_150	-1,143	0,0365	248	-1,265	-1,022	bcdefghijklm
24h	Purple_75	-1,142	0,0365	248	-1,263	-1,02	bcdefghijklm
0h	S1_1:8	-1,141	0,0365	248	-1,262	-1,019	bcdefghijklm
24h	N_150	-1,141	0,0365	248	-1,262	-1,019	bcdefghijklm
24h	N_37,5	-1,14	0,0365	248	-1,262	-1,019	bcdefghijklm
0h	N_75	-1,136	0,0365	248	-1,258	-1,015	bcdefghijklm
24h	N_18,75	-1,136	0,0365	248	-1,257	-1,014	bcdefghijklm
48h	N_150	-1,135	0,0365	248	-1,256	-1,013	bcdefghijklm
24h	Purple_37,5	-1,132	0,0365	248	-1,253	-1,011	fghijklm

24h	S1_1:1	-1,131	0,0365	248	-1,253	-1,01	bcdefghijklm
24h	K_75	-1,131	0,0365	248	-1,252	-1,009	bcdefghijklm
24h	S1	-1,13	0,0365	248	-1,251	-1,009	dejklm
24h	K_37,5	-1,129	0,0365	248	-1,251	-1,008	bcdefghijklm
24h	K_18,75	-1,129	0,0365	248	-1,251	-1,008	bcdefghijklm
24h	S1_1:8	-1,124	0,0365	248	-1,246	-1,003	bcdefghijklm
0h	K_37,5	-1,124	0,0365	248	-1,245	-1,003	bcdefghijklm
24h	S1_1:4	-1,123	0,0365	248	-1,245	-1,002	bcdefghijklm
24h	Mock	-1,121	0,0365	248	-1,242	-0,999	fghijklm
24h	K_150	-1,12	0,0365	248	-1,242	-0,999	fghijklm
24h	Purple_18,75	-1,117	0,0365	248	-1,239	-0,996	gikm
0h	K_75	-1,116	0,0365	248	-1,237	-0,994	fghijklm
0h	N_37,5	-1,097	0,0365	248	-1,218	-0,975	lm
0h	Mock	-1,092	0,0365	248	-1,214	-0,971	lm
0h	N_18,75	-1,078	0,0365	248	-1,2	-0,957	lm
0h	K_150	-1,07	0,0365	248	-1,191	-0,948	lmn
48h	Purple_75	-0,867	0,0365	248	-0,989	-0,746	no
48h	N_75	-0,677	0,0365	248	-0,798	-0,555	op
48h	Purple_37,5	-0,599	0,0365	248	-0,72	-0,478	pq
48h	S1	-0,515	0,0365	248	-0,636	-0,394	pqr
48h	S1_1:1	-0,49	0,0365	248	-0,612	-0,369	pqr
48h	Purple_18,75	-0,484	0,0365	248	-0,605	-0,362	pqr
48h	N_300	-0,475	0,0365	248	-0,597	-0,354	pqr
48h	S1_1:4	-0,45	0,0365	248	-0,571	-0,329	qr
48h	S1_1:8	-0,448	0,0365	248	-0,569	-0,326	qr
48h	N_150	-0,431	0,0365	248	-0,552	-0,31	qr
48h	K_1200	-0,41	0,0365	248	-0,531	-0,288	qr
48h	K_600	-0,394	0,0365	248	-0,515	-0,272	qr
48h	K_150	-0,393	0,0365	248	-0,514	-0,271	qr
48h	K_300	-0,388	0,0365	248	-0,509	-0,266	r
48h	Mock	-0,384	0,0365	248	-0,505	-0,262	r

[1] estimated marginal means, [2] standard error, [3] digit of freedom, [4] lower confidence interval, [5] upper confidence interval, [6] statistical group

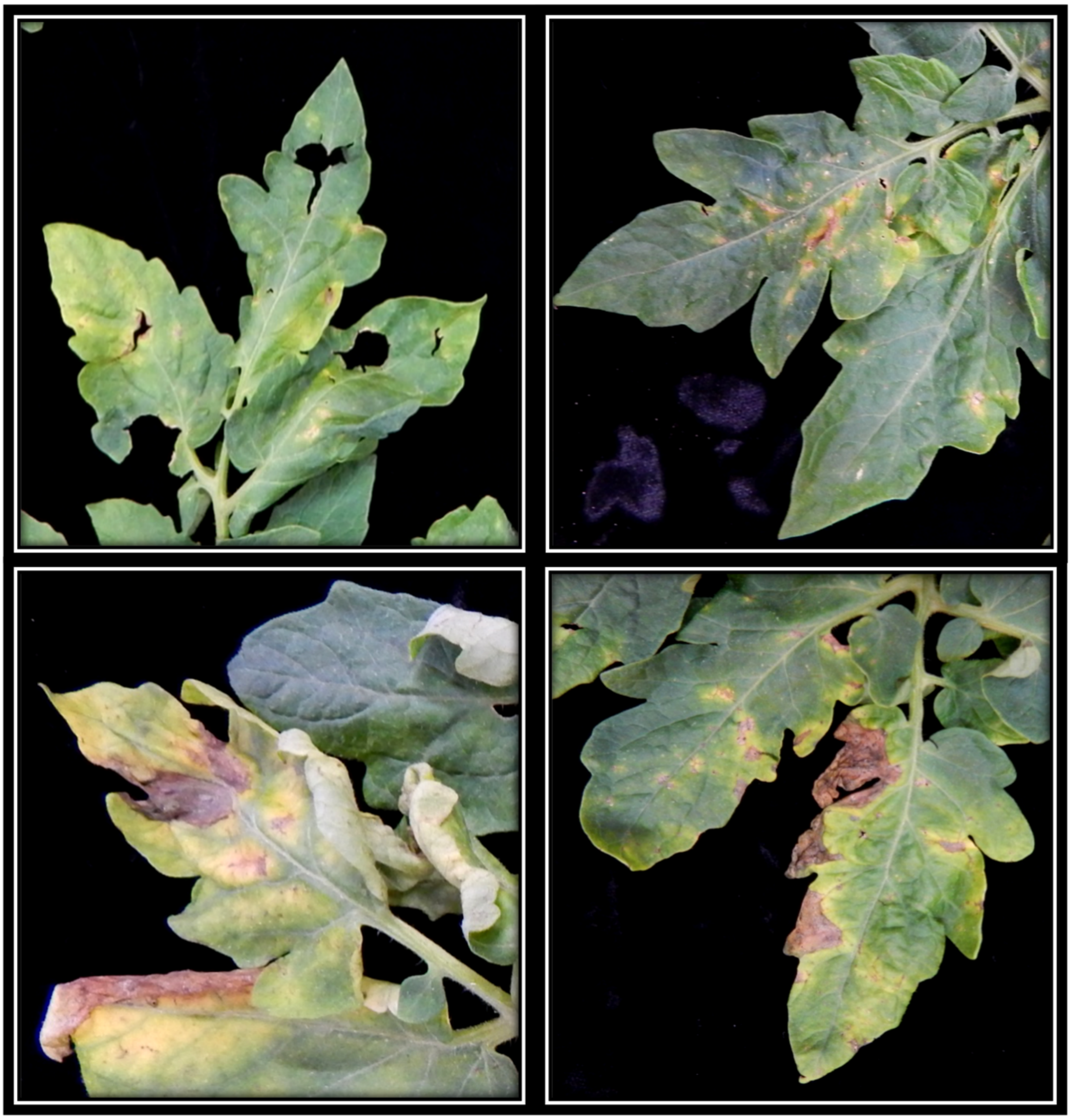

Figure S2. Symptoms on tomato leaves 30 days post infection with *Xanthomonas campestris* pv. *vesicatoria* by spraying.
